# Myocardial Contrast Echocardiography in the Evaluation of Hypertensive Heart Disease

**DOI:** 10.4021/cr93w

**Published:** 2011-11-20

**Authors:** Ernest C. Madu, Chiranjivi Potu, Dainia Baugh, Edwin Tulloch-Reid

**Affiliations:** a Department of Medicine, Division of Cardiovascular Medicine, Heart Institute of the Caribbean, Kingston, Jamaica; b Center of Excellence for Cardiovascular Medicine and Sports Physiology, University of Technology, Kingston, Jamaica

**Keywords:** Contrast Echocardiography, Left ventricular hypertrophy, Coronary flow reserve

## Abstract

Myocardial contrast echocardiography (MCE) has an established role in left ventricular assessment by improving the ventricular opacification and endocardial border definition especially in patients with sub-optimal echocardiographic images. With advances in cardiac ultrasound imaging technology and the development of new contrast agents, the clinical utility of this technique has greatly expanded to include assessment of coronary reperfusion in the setting of acute myocardial infarction, determination of myocardial viability within infarct zones as well as assessment of coronary microcirculation and flow reserve in patients with microvascular coronary disease. Improvements in image quality with intravenous contrast agents can facilitate image acquisition and enhance delineation of regional wall motion abnormalities at peak levels of exercise. Numerous studies have confirmed the clinical utility of contrast enhancement during echocardiographic studies, particularly in patients undergoing stress testing. In this paper, we explore the evidence in support of MCE and its potential clinical applications. Our review aims to summarize (1) the basic principles of myocardial contrast echocardiography including recent advances in the ultrasound technology and contrast agents (2) its clinical applications in the diagnosis of cardiovascular diseases and finally, (3) its potential role in risk stratification and assessment of microvascular perfusion in patients with hypertensive heart disease.

## Introduction

Recent developments in contrast agents and acoustic technology have opened new opportunities in the clinical applications of myocardial contrast echocardiography (MCE). Beyond evaluation of cardiac structures and function, MCE can provide much needed insight into myocardial perfusion and thus permit a better understanding of pathophysiologic mechanisms in patients with abnormal LV function. This role appears to have specific advantage in patients with microvascular disease particularly in the absence of concomitant epicardial coronary artery disease. In this paper we briefly describe recent advances in the technology of MCE and potential application of these new advances in the evaluation of patients with hypertensive heart disease.

## Rationale for Myocardial Contrast Enhancement

Early use of contrast in echocardiography was necessitated by the need to better visualize cardiac anatomy. Since the initial description of echocardiographic contrast effect in the aortic root by Gramiak and Shah more than four decades ago [1], significant advances in both contrast technology and instrumentation have enabled clinicians and researchers to extend the application of contrast echocardiography to the study of left ventricular systolic and diastolic functions, valvular flows, intrapulmonary and intracardiac shunts, intracardiac clots and masses as well as in intramyocardial blood flows and perfusion [2-5]. Major initial limitations imposed by the relatively large size and instability of the microbubbles have mainly been overcome by the relatively stable and smaller microbubbles of the later generation contrast agents (Table 1). These newer contrast agents are capable of surviving transpulmonary transit, thus allowing for opacification and evaluation of the left ventricle [6]. Myocardial contrast echocardiography is based on the scientific principle that air filled microbubbles produce contrast effect and that air in microbubbles possess acoustic characteristics (density, speed propagation and absorption of sound waves) different from the surrounding solution, thus producing ultrasonic contrast [7, 8].

**Table 1:** Contrast Agents for Contrast Echocardiography

Name	Gas	Mean diameter (Micro meters)	Concentration .ml^-1^	Comments
Levovist	Air	1.2	1.2-2.0 .10^8^	Available in many countries but not in USA
Albunex	Air	4.3	0.5 .10^9^	For LV opacification
Imagent	Perfluorohexane	5.0	0.5 .10^8^	For LV opacification
Optison	Perflutren	3.0-4.5	5.0-8.0 .10^8^	Available in USA and Europe for LV opacification
Sonazoid	Perflubutane	2.4-2.5	0.3 .10^9^	Available in Japan for Liver opacification
Definity	Octafluropropane	1.1-3.3	1.2 .10^10^	Available in USA, Europe for LV opacification
Sonovue	Sulphur hexafluoride	2.5	5.0 .10^8^	Available in Europe for LV Opacification
Cardiosphere	Nitrogen	3.0	2.0 - 5.0 .10^8^	Under FDA review for MCE
Imagify	Decafluorobutane	2.3	Gas 260 ± 25 micro g.ml^-1^ of reconstituted produc	Under FDA review for MCE

FDA: Food and Drug Administration.

To a large extent the ability of contrast agents to opacify and hence allow the evaluation of the LV has been made possible by the introduction of sonication by Feinstein et al in 1984 [6]. Sonication involves the exposure of solution to ultrasound resulting in the formation of small, stable, uniform, non-energy dependent microbubbles capable of crossing the pulmonary capillaries into the LV. This process involves 4 steps: (a) formation of microcavities intrinsic to the solution; (b) development of large vibrant microbubbles; (c) disintegration of microbubbles; and (d) formation of stable forms of the microbubbles. This process has been aided by significant improvements in instrumentation technology, notably, second harmonic and transient response imaging [9].

## Role of Myocardial Contrast Echocardiography in Clinical Practice

The availability of stable microbubbles and the current performance limits of ultrasound imaging and Doppler techniques have encouraged the growth of contrast echocardiography in clinical practice. By increasing the signal to noise ratio, ultrasound contrast agents have greatly improved the sensitivity and specificity of diagnostic ultrasound imaging. Routine clinical applications have been found in the study of myocardial ischemia during functional stress testing, endocardial border delineation and evaluation of valvular flows. Perhaps the most exciting potential clinical utility of contrast echocardiography is in the evaluation of microvascular flows and “no-reflow” phenomenon.

Chamber opacification and improved endocardial border delineation allow for accurate assessment of LV volume and hence a more precise estimation of cardiac function. In phase III clinical trials, Albunex^®^, an earlier generation contrast agent was found to be effective in achieving adequate LV opacification in 81% of cases and improving LV endocardial definition in 83% of patients [10]. Crouse et al [11] have also demonstrated that investigator confidence in assessing LV wall motion was improved by 80% following administration of intravenous albumin. Improvement in endocardial border delineation was noted in over 90% of patients. These finding have since been translated into clinical application in several studies evaluating wall motion during stress echocardiography with ultrasound contrast enhancement. Markowitz et al [12] have demonstrated that 77% of poorly visualized myocardial segments during dobutamine stress echocardiography had improved visualization following intravenous contrast administration. When exercise stress echocardiography interpretations were evaluated for accuracy, Marwick et al [13] reported that poor image quality potentially accounted for up to 43% of incorrectly read studies. This finding probably explains the beneficial role of contrast enhancement in improving the sensitivity and specificity of stress echocardiography in the evaluation of coronary artery disease.

Albunex^®^ was the first commercially available contrast agent. Developed by sonication of 5 % albumin solution, Albunex^®^ has excellent myocardial opacification on intracoronary injection but does not opacify left ventricle after intravenous injection [14]. After intravenous administration of albunex, the air in the microbubbles being highly diffusible, leaks out as they transit the pulmonary circulation leading to a reduction in the size of the microbubbles. The acoustic backscatter from a bubble is related to the sixth power of its radius with smallest change in the microbubble size resulting in large decrease in ultrasound backscatter resulting in poor LV opacification [15]. The significant limitations imposed by the albunex as an ideal contrast agent have been largely replaced by the development of newer generation contrast agents such as EchoGen^®^, FS069 (Optison^®^) and DMP-115 (Definity)^®^).The micro-bubbles in these newer generation contrast agents contain larger molecular weight gases with low diffusion capacity thus creating stable bubbles which do not dissolve in blood. They enter the myocardium intact permitting effective backscatter and ultimately satisfactory visualization of the left ventricular cavity as well as the myocardium after intravenous injection thus allowing one to directly evaluate myocardial perfusion [16, 17]. This property has made possible the use of contrast agents in the study of microvascular integrity, ventricular remodeling, “no-reflow” phenomenon [18], collateral flow and viability [19, 20] as well as post-infarct or ischemia prognosis [2, 3, 21].

Perflenapent emulsion (EchoGen^®^), a phase-shift colloid belongs to the newer class of fluorocarbon based ultrasound contrast agents offering better endocardial border delineation and LV opacification. Clinical investigations have thus far shown promise with EchoGen and other new generation contrast agents (e.g., Optison and Definity) in visualization and localization of myocardial perfusion defects at rest by producing a negative contrast effect. Unlike albumin, a less stable contrast agent, the microbubbles produced by EchoGen, Optison and Definity persist in the LV for much of systole and are able to make multiple passes through the portal vein. The microbubbles are small, uniform and stable and thus survive transpulmonary transit, allowing for circulation in the vascular system and enabling imaging of small blood vessels and tissues [22].

The persistence of the contrast effect during a significant portion of the cardiac cycle and lack of significant attenuation at doses capable of producing myocardial enhancement permits interrogation in multiple echocardiographic views. In clinical trials evaluating EchoGen^®^, improvements in endocardial border delineation and cardiac function assessment have been demonstrated. EchoGen^®^ was associated with improvement in blood pool contrast enhancement, facilitation of endocardial border delineation and visualization of valvular blood flows, improvement in the quality of wall motion abnormalities and estimation of systolic function [23].

The role of MCE in the setting of suspected acute myocardial infarction has been well established. Kaul et al [24] in a study involving 203 patients demonstrated the superior role of MCE in the evaluation of acute coronary syndrome in the emergency department compared with routine clinical evaluation. Tong et al [25] have shown that the determination of regional function and myocardial perfusion with MCE is superior to Thrombolysis in Myocardial Infarction (TIMI) score for diagnosis and prognostication in patients presenting to the emergency department with chest pain and a nondiagnostic electrocardiogram. The incremental value of determining the regional function and myocardial perfusion with MCE is further established by Rinkevich et al [26] in a study involving the 1017 patients being evaluated for chest pain in the emergency department. In acute myocardial infarction, MCE can define the risk area [27] confirm the success of the reperfusion [28, 29] and residual infarct size [3] (via no re-flow phenomenon). It can also be used to assess the presence and extent of collateral perfusion during acute coronary occlusion and its impact on myocardial viability [30, 31]. MCE has also been used successfully for the detection of stable chronic coronary artery disease in the absence of prior infarction [32, 35].

## Potential Role of Contrast Echocardiography in Hypertensive Heart Disease

**Figure 1:**
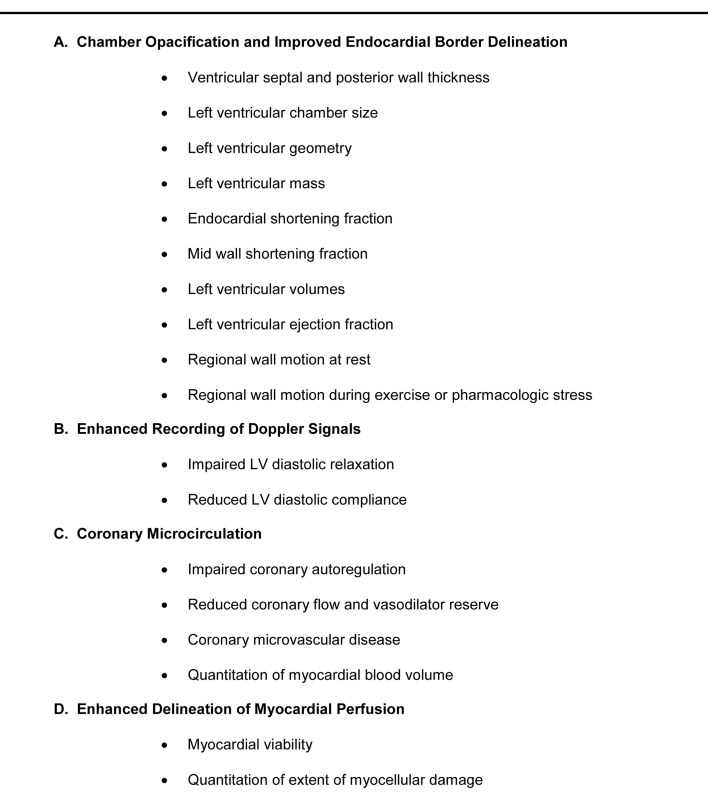
Application of contrast echocardiography in patients with systemic hypertension and hypertensive heart disease.

Hypertensive heart disease comprises a wide spectrum of adverse alterations in cardiovascular structure and function attributable to hypertension and which predispose patients to premature morbidity and mortality. This spectrum includes left ventricular hypertrophy, left atrial enlargement, aortic root dilatation, aortic dissection, vascular hypertrophy, reduced arterial vascular compliance, asymptomatic and symptomatic LV systolic dysfunction, diastolic heart failure, coronary microvascular disease, atrial and ventricular arrhythmias and sudden cardiac death [36-39]. By convention, obstructive epicardial coronary artery disease, although a known complication of hypertension is not considered in the spectrum of hypertensive heart disease. Echocardiography has been ideal in the assessment of the majority of these conditions. Contrast echocardiography provides a potential unique opportunity for further assessment of these conditions in 4 main scenarios as shown in (Fig. 1)

Left ventricular hypertrophy is a common cardiac complication of chronic hypertension. It is also a powerful, independent marker of increased risk for cardiovascular mortality and death from all causes in both men and women [40-42]. Echocardiography is more sensitive than ECG and may detect LVH in 13-24% of men and 20-45% of women with mild hypertension in whom LVH is virtually absent in ECG [43]. Although echocardiography detects LVH in fewer than 12-30% of unselected patients with mild, uncomplicated hypertension, it is estimated that up to 60% of adult hypertensive patients referred to tertiary care and specialized hypertension centers and 90% of patients with chronic severe or malignant hypertension may have LVH [44, 45]. An important limitation in the routine assessment of LVH is poor endocardial definition rendering the primary measurements of wall thicknesses and chamber dimension imprecise. Chamber opacification and improved endocardial border delineation during echocardiographic contrast injection will help improve primary measurements and facilitate reliable determination of LV mass and chamber geometry.

Chamber opacification and enhanced endocardial border definition will also facilitate reliable calculation of left ventricular ejection fraction and systolic performance. These data are essential to the proper diagnosis as well as prognosis in hypertensive heart disease. Even minimally depressed LV ejection fraction is important to confirm in the hypertensive patient. It represents an important phase in the spectrum of cardiac damage from hypertension. Left untreated, even minimally depressed systolic function invariably progresses to symptomatic heart failure because of the activation of neurohormones that further hasten adverse ventricular remodeling. Survival is significantly reduced (15-18%) at 2 years in patients with asymptomatic LV systolic dysfunction [46, 47]. Symptomatic heart failure with significant reductions in LV ejection fraction is a common finding in hypertensives. Considered as the sole etiology, hypertension accounts for a significant proportion of heart failure etiology in both men and women [48]. In a report on the 14-year (mean) follow-up of 5143 subjects without previous heart failure, hypertension was present in 91% of the 394 subjects who developed heart failure [49]. In 59% of women and 39% of men, hypertension was the primary etiology for the incident heart failure [49]. All recent guidelines for the evaluation and management of heart failure call for objective assessment of LV ejection fraction [50-53]. Echocardiography has been the preferred modality. In individuals with suboptimal echocardiograms, the use of echocardiographic contrast may obviate the need to refer for a second study (such as radionuclide ventriculography) for the non-invasive assessment of LV function.

In hypertensive patients with symptoms of heart failure but preserved LV systolic function and no evidence of valvular, pericardial or primary myocardial disease, LV diastolic dysfunction is an important condition and commonly, the culprit. In addition to confirming a normal LV ejection fraction and excluding structural heart disease, the demonstration of abnormal trans-mitral Doppler spectrum is important for echocardiographic diagnosis. However, a pseudo-normal Doppler spectrum may be seen when abnormalities of relaxation, compliance and restrictive physiology coexist. The use of Valsalva maneuver and a careful recording of the pulmonary vein Doppler spectrum can help unmask diastolic dysfunction [54-56]. In patients with suboptimal imaging and recording of the pulmonary venous spectrum, the use of echocardiographic contrast allows this useful information to be obtained from the transthoracic echocardiogram.

## Contrast Echocardiography in the Coronary Microcirculation and Myocardial Perfusio

Myocardial contrast echocardiography is an ideal imaging tool for the assessment of coronary microcirculation [57]. MCE can define vessels with a diameter < 10 µm and thus is superior to coronary angiography (which can define vessels >100 µm in diameter) in demonstrating collateral circulation [58, 59].This potential application of myocardial contrast enhancement in the study of coronary microcirculation and perfusion in the beating heart has heightened the interest in MCE in the study of hypertensive heart disease.

Microvascular flow may be abnormal in hypertensive heart disease especially in the setting of LVH, and may indeed produce angina symptoms even in the absence of significant epicardial coronary artery disease. By defining the region of abnormal microvascular flow or perfusion with MCE, it is possible to quantify the extent of myocellular damage [29, 60]. The extent of microvascular perfusion is an index of myocardial viability and can be used to further assess and risk stratify patients with hypertensive hypertrophic cardiomyopathy, particularly in the absence of significant epicardial coronary artery disease. Because of the limitation of coronary angiography in assessing flows in minute vessels, it is unable to predict microvascular perfusion patterns. The ability of MCE to accurately define relative myocardial perfusion has been successfully employed in assessing ischemia/reperfusion, myocardial recovery following percutaneous coronary interventions and also by surgeons during anterograde cardioplegia delivery through the cross-clamped aorta [58, 61, 62] or to assess adequacy of revascularization in the operating room.

Myocardial contrast echocardiography is able to demonstrate, not only the presence or absence of microvascular perfusion, but can quantify myocardial blood volume and microvascular reserve [63, 64]. When applied to patients with hypertensive heart disease, this information can be used for risk stratification of patients and also in quantification of risk as well as the effects of different risk management strategies on prognosis. Analogous to the application of nuclear perfusion techniques in patients with CAD, MCE can potentially be used to study rest and hyperemic microvascular flows in patients with hypertensive heart disease. The spatial extent and amount of myocardium susceptible to abnormal microvascular flows can form the basis for risk stratification, management and prognostication of patients with hypertensive heart disease, particularly in the absence of significant epicardial coronary artery disease.

Coronary blood flow reserve (expressed as the ratio of hyperemic to basal flow) is dependent on the inherent vasodilatory properties of the coronary microvasculature [58] and blood viscosity. Abnormal coronary flow reserve can occur in patients in with hypertensive heart disease in the absence of epicardial coronary artery disease. Several studies have documented abnormal coronary blood flow reserve in patients who have risk factors for CAD in the absence angiographically evident coronary artery disease [65]. Abnormal coronary blood flow reserve in hypertensive heart disease is primarily due to abnormal microvascular flow reserve which is impaired in patients with hypertensive heart disease. The degree of impairment is related to the severity of hypertensive heart disease. Changes in microvascular flow reserve occur predominantly at the level of the capillaries. Hypertension causes damage to the capillaries in the myocardium resulting in either anatomical or functional loss. Hypertension induced damage to the capillaries (anatomical or functional) result in the recruitment of less number of capillaries during exercise and explains the reduced coronary flow reserve and the resultant episodes of recurrent exercise induced ischemia in the absence of coronary stenosis [66]. There is currently no universally accepted non-invasive diagnostic modality for assessment of microvascular coronary flow reserve. Pharmacologically induced hyperemic flows can be directly assessed by MCE and gives a physiologically appropriate evaluation of microvascular flow reserve and collateral circulation [67-70]. Proper serial evaluation of microvascular flow reserve in patients with hypertensive heart disease can potentially be performed rapidly and noninvasively utilizing MCE. Infact, in a study, using peripherally administered contrast agent, Mills et al [69], demonstrated that myocardial contrast echocardiography with harmonic imaging, was useful in mapping the spatial distribution and time course of coronary collateral development in an ischemic bed. This observation suggests that MCE could become a potentially viable modality for the serial evaluation of time course of disease progression and response to therapeutic interventions. The portability of this technique, absence of radiation exposure and the ease of performance of MCE compared to coronary arteriography and radionuclide procedures, makes it a preferred modality for serial evaluation of disease and intervention. Additionally, coronary arteriography and competing radionuclide techniques such as SPECT and PET imaging are limited in spatial resolution and thus suboptimal for evaluation of microvascular disease or time course changes related to disease progression or intervention. MCE can also be performed repetitively without the need for invasive coronary manipulation or radiation exposure thus making it the ideal technique for safely monitoring the progression of disease or improvement resulting in various intervention strategies.

## Future Prospects and Applications

Currently, only 2 contrast agents (Albunex and Optison) are approved for use in the USA by the Food and Drug Administration (FDA). These agents are approved for the indication of left ventricular opacification and enhanced endocardial border delineation, but not for myocardial perfusion. In line with earlier observations by Marwick et al [13], review of phase III clinical trial data on the use of Optison and other contrast agents have revealed significant qualitative improvement in about 50% of the images [71]. The use of contrast agents particularly in those with suboptimal echo study, therefore, could significantly enhance the quality of ultrasound images, improve diagnostic accuracy, reduce downstream testing and associated costs, and potentially improve patient outcome.

Perhaps, the most promising future application of myocardial contrast echocardiography is in the routine clinical evaluation of myocardial perfusion, coronary, and intramyocardial blood flow. It is expected however that, that with the level of interest and research currently in progress, our understanding of ultrasound-contrast interaction will witness an exponential growth and would spur refinements in machine technology that ultimately will lead to routine clinical application of this technique in patients with microvascular disease such as those with Hypertensive Heart Disease.

## Conclusion

MCE will undoubtedly continue to gain prominence in the evaluation and management of cardiovascular diseases. It is uniquely positioned to fill a major void in the non-invasive assessment of microvascular flows particularly in patients with hypertensive heart disease and associated co-morbidities. With continued progress and refinements in acoustic technology and contrast agents, these potential applications will soon be translated to routine clinical use.
